# Sex differences in thermal detection and thermal pain threshold and the thermal grill illusion: a psychophysical study in young volunteers

**DOI:** 10.1186/s13293-017-0147-5

**Published:** 2017-09-01

**Authors:** Beate Averbeck, Lena Seitz, Florian P. Kolb, Dieter F. Kutz

**Affiliations:** 1 Department of Physiology, University of Munich, Munich, Germany; 2 Institute of Human Movement Science and Health, Faculty of Behavioral and Social Science, Chemnitz University of Technology, Chemnitz, Germany; 3 Department of Physiology, Biomedical Center Munich (BMC), University of Munich, Planegg-Martinsried, D-82152 Germany

**Keywords:** Sex differences, Psychophysics, Thermal thresholds, Thermal grill illusion, Thermal pain, Cold sensitivity, Cold receptors

## Abstract

**Background:**

Sex-related differences in human thermal and pain sensitivity are the subject of controversial discussion. The goal of this study in a large number of subjects was to investigate sex differences in thermal and thermal pain perception and the thermal grill illusion (TGI) as a phenomenon reflecting crosstalk between the thermoreceptive and nociceptive systems. The thermal grill illusion is a sensation of strong, but not necessarily painful, heat often preceded by transient cold upon skin contact with spatially interlaced innocuous warm and cool stimuli.

**Methods:**

The TGI was studied in a group of 78 female and 58 male undergraduate students and was evoked by placing the palm of the right hand on the thermal grill (20/40 °C interleaved stimulus). Sex-related thermal perception was investigated by a retrospective analysis of thermal detection and thermal pain threshold data that had been measured in student laboratory courses over 5 years (776 female and 476 male undergraduate students) using the method of quantitative sensory testing (QST). To analyse correlations between thermal pain sensitivity and the TGI, thermal pain threshold and the TGI were determined in a group of 20 female and 20 male undergraduate students.

**Results:**

The TGI was more pronounced in females than males. Females were more sensitive with respect to thermal detection and thermal pain thresholds. Independent of sex, thermal detection thresholds were dependent on the baseline temperature with a specific progression of an optimum curve for cold detection threshold versus baseline temperature. The distribution of cold pain thresholds was multi-modal and sex-dependent. The more pronounced TGI in females correlated with higher cold sensitivity and cold pain sensitivity in females than in males.

**Conclusions:**

Our finding that thermal detection threshold not only differs between the sexes but is also dependent on the baseline temperature reveals a complex processing of “cold” and “warm” inputs in thermal perception. The results of the TGI experiment support the assumption that sex differences in cold-related thermoreception are responsible for sex differences in the TGI.

**Electronic supplementary material:**

The online version of this article (doi:10.1186/s13293-017-0147-5) contains supplementary material, which is available to authorized users.

## Background

Evidence suggests women and men experience and report pain differently. The most pronounced sex differences have been found for heat pain, with females showing lower heat pain threshold, tolerating less thermal heat and perceiving hot temperatures as more painful and more unpleasant than males [1–6]. Sex differences in cold pain as well as in thermal non-painful sensation have been described more rarely in the literature and the existing studies report higher sensitivity of females compared with males [2, 4, 7, 8]. Sex differences in the thermal grill illusion (TGI), a phenomenon reflecting crosstalk between the thermoreceptive and nociceptive systems, have not been investigated so far.

The TGI, first reported by Thunberg in 1886, is generated by pairing innocuous warm and cold temperatures. This leads to a sensation of strong, but not necessarily painful, heat often preceded by transient cold [9]. Several studies indicate that the TGI is a very complex phenomenon that is generated by central higher order processing and reveals a relationship between the thermoreceptive and nociceptive systems [10–23]. Thus, sex differences in thermoreception and thermal pain perception may be related to sex differences in the TGI.

The aim of the present study was to determine sex differences in the TGI by testing 136 undergraduate medical students. We recorded qualities and intensities of sensation evoked by a 20/40 °C thermal grill stimulus in comparison to a uniform cold (20 °C) or warm (40 °C) stimulus in order to analyse sex differences in the changes of sensation evoked by thermal grill stimulation. In addition, we determined sex differences in thermal detection and thermal pain threshold in 1252 undergraduate students of medicine and dentistry, by retrospectively analysing quantitative sensory testing (QST) data that had been collected in student laboratory courses. QST data included cold and warm detection thresholds at different baseline (adaptation) temperatures between 20 and 40 °C. This allows further investigation of thermal sensation circuitries as earlier studies have analysed thermal detection thresholds only at baseline temperatures around the neutral/comfort zone of 32 °C [24, 25].

After finding sex differences in TGI and QST data, the objective was to test the hypothesis of a sex-dependent correlation of the TGI with the subject’s thermal sensitivity and/or thermal pain sensitivity. Therefore, we correlated the TGI with cold or warm sensation and, in addition, with thermal pain sensitivity.

## Methods

### Subjects

The thermal grill experiments were performed in a group of 136 medical undergraduate students of the Ludwig-Maximilians University Munich (58 males and 78 females, aged 20–30 years). Volunteers were recruited by signing a list available in the student laboratories. This list included all information about the purpose of the study including the aim, i.e. the investigation of sex differences in thermoreception. On the day of the experiment, the subjects gave consent to participate in the experiments.

The retrospective analysis of thermal sensation data included 12,874 records of QST measurements from 1252 students (776 females and 476 males, mean age 22 ± 3 years (median interquartile range)). The data were collected during neurophysiological laboratory courses for medical and dental undergraduate students at the Ludwig-Maximilians University Munich (Germany) in the years 2007–2011. Students were informed that data of thermal sensitivity were to be gathered from healthy subjects and that the data were later to be analysed anonymously to generate comprehensive results for instruction and publication purposes. Six or seven students of each class of around 20 students volunteered to undergo the non-invasive tests; the others performed the acquisition of data or other tasks. Volunteers gave consent to participate in the experiments before the start of the tests. The analysis was performed with the permission of the local ethics committee of the Ludwig-Maximilians University of Munich.

For analysing correlations of thermal pain threshold with the TGI, all values were determined in one experimental session for each of 40 (20 female and 20 male) medical students. The recruitment of these subjects (aged 20–30 years) who were not part of the 136 cohort was the same as described above for the thermal grill experiments. On the day of the experiment, the subjects gave consent to participate in the experiments. The whole study (all three experiments) complied with the guidelines established by the Declaration of Helsinki and was approved by the local ethics committee of the Ludwig-Maximilians University Munich for experiments involving human subjects.

### Equipment and experimental protocol

The thermal grill experiments were performed by an investigator. The subjects were naïve with respect to the “illusion phenomenon”. They were informed about the rating procedure and assured of the harmlessness of all stimulation parameters. For stimulation, subjects placed the palmar surface of the right hand on the thermal grill that was fixed to a table. The setup of the thermal grill device has been described elsewhere [19]. Briefly, the thermal grill consists of 15 bars (tubes) that are perfused with warm or cold water. The temperatures tested were 20, 40, and 20 °C alternating with 40 °C (thermal grill stimulus). A second thermal grill with all bars (tubes) held at 32 °C was used as a control to establish a baseline temperature of the skin immediately prior to each thermal stimulus trial.

To examine sensations associated with grill stimulation, the hand was first placed on the 32 °C reference grill surface for 20 s. The hand was then exposed to either a uniform 20° or uniform 40 °C stimulus or the interleaved 20/40 °C (grill) stimulus for 20 s. Each stimulus was presented three times with a minimum inter-stimulus interval of 5 min. After each stimulus presentation, subjects were asked to specify their evoked perception by using the descriptors “warm/heat, cold, unpleasantness, pain, burning, stinging and prickling”. Then, the subjects rated the intensities of their sensations using numeric rating scales (NRS) from 0 to 100 to rate the “thermal intensity” of (a) their cold sensation and (b) their warm sensation. The scale anchors were “0 = neutral” and “100 = worst cold or worst warm/hot”, respectively, and along one side of the scale there were three additional descriptors indicating that the subject should rate intensities of perceived coldness or warmth/heat. The scale was numbered from 0 to 100 in increments of 10. In addition, subjects rated perceived pain and unpleasantness on numeric scales from 0 to 100, also numbered from 0 to 100 in increments of 10 and with the anchors “0 = no pain or no unpleasantness” and “100 = worst pain or as unpleasant as can be imagined”.

QST measurements were self-performed by the students in groups of two or three. The students were informed about the procedure of the measurements but received no prior training. In each group, one student served as subject and the other(s) operated the computer and recorded the data. For stimulation, a computerized thermotest device TSA 2001-II Neurosensitive Analyser (MEDOC, Ramat Yishai, Israel) was used with a standard 30 × 30 mm thermode. The method of limits was employed [26] and the rate of temperature change was 1.5 °C/s. The cut-off temperatures were 0 and 50 °C, respectively. During the experiment, the subject was not able to see the computer screen.

Cold and warm detection thresholds (CDT and WDT, respectively) were measured at different baseline temperatures of the thermal ramp stimulus with the thermode affixed with a Velcro strip to the ventral surface of the forearm near the wrist. Table 1 shows the sequence of measurements at different baseline temperatures in the range of 20–40 °C resulting in different values of CDT_X°C_ and WDT_X°C_. The subject signalled that a threshold had been reached by pressing a button, at which point the temperature change of the thermode was halted, the direction reversed and the temperature returned to the respective baseline temperature. Subjects were instructed to press the button as soon as they detected a change of the temperature (“Press the response button immediately when you perceive a change of the thermode temperature, i.e. warmer during WDT tests or colder during CDT tests”). Thresholds of five consecutive runs were averaged to determine CDT_X°C_ and WDT_X°C_.

**Table 1:** Sequence of measurements at different baseline temperatures in the range of 20–40 °C

Recording sequence	Baseline temperature	Sequence of detection thresholds
1st	30 °C	WDT_30 °C_, CDT_30 °C_
2nd	35 °C	CDT_35 °C_, WDT_35 °C_
3rd	25 °C	WDT_25 °C_, CDT_25 °C_
4th	40 °C	CDT_40 °C_, WDT_40 °C_
5th	20 °C	WDT_20 °C_, CDT_20 °C_

For measuring cold and heat pain threshold (CPT and HPT), the thermode was placed on the subject’s skin so as to stimulate the thenar eminence. The baseline temperature was 32 °C. The instruction was as follows: Indicate by pressing a button the occurrence of a painful or unpleasant sensation of heat or cold, respectively. Thresholds of five consecutive runs were averaged to determine CPT and HPT.

Data used for correlation analyses of the TGI with thermal pain threshold were obtained in an additional experiment that was carried out by an investigator. The TGI and CPT and HPT were measured in 40 subjects (20 female and 20 male medical students) who had been trained in QST measurements. The subjects were naïve with respect to the “illusion phenomenon” according to the thermal grill experiment with 136 subjects. The thermal grill and QST parameters were the same as described above.

### Statistical analysis

Statistical analysis was performed using the program SPSS Statistics Version 22, IBM, Chicago, IL, USA. With respect to the thermal grill experiments, Kolmogorov–Smirnov test revealed that for NRS ratings of thermal sensation and for thermal threshold, data values were not normally distributed. Therefore, the median as well as the first and third quartiles (boxes) and range (error bars) were used for data description and Friedman’s ANOVA with post hoc testing (Wilcoxon’s signed-rank test) was employed for statistical analysis of the thermal grill-evoked sensations. To assess sex differences in the qualities of the thermal grill-evoked sensations, the Mann-Whitney *U* test was performed. Differences in sensations evoked by the uniform cold (20 °C) or warm (40 °C) stimulus and the thermal grill (20°/40 °C interleaved) stimulus are presented as means ± standard error of the mean (SEM; *Δ* values) for male and female subjects separately. For analysing sex differences in the intensities of grill-evoked sensations, Student’s *t* test for unpaired samples was performed. Linear regression analyses were carried out to evaluate the effects of thermal sensation and thermal pain sensation on the effects of the thermal grill-evoked warmth/heat and coldness. To assess dependency between two variables of non-normally distributed data, Spearman’s correlation coefficient (*ρ*) was calculated. A Bonferroni-type adjustment was made for multiple correlation analyses. *P* value < 0.05 was considered to indicate a statistically significant difference and is indicated by an asterisk (*) in the tables and figures.

To assure the quality of the retrospectively analysed QST data, the variances of all 12.874 records were analysed. The variances were distributed in the range 0.00–260.26. The distribution of variances is characterized as follows: median 0.280, interquartile range (IQR) 1.430, mean 2.577, and standard deviation (SD) 9.565. Skewness was reduced calculating an upper criterion (Eq. 1), following McGill and colleagues [27]

1 $$ \mathrm{crit}\kern0.5em =\kern0.5em \mathrm{median}+1.5\times \mathrm{IQR} $$

Any record with a variance above a crit value of 2.425 was excluded from further analysis. In addition, records with incomplete or implausible data were excluded, i.e. data lacking the information about sex or age or data obtained using incorrect stimulation sites. Some 9940 records remained for further analysis. Following the suggestions by Rolke and colleagues [2], all records were logarithmized (base 10) before being subjected to any statistical test. Sex differences were analysed post hoc using Tukey’s honest significant difference test [28]. Analyses were performed using the language for statistical computing R [29].

For analysis of multimodal distribution of CPTs, the CPT data were fitted by a Gaussian mixed model using the R-program AdaptGauss [30] (Additional file 1). For calculating the probability density function (PDF), the median of five repetitions of the CPT test of each subject was estimated and rescaled for stimulus intensity and a log transformation was subsequently carried out. The PDF was calculated for all subjects participating in the CPT measurements (*N* = 296).

## Results

### Thermal grill illusion (TGI)

Thermal grill-evoked sensations were analysed in 136 subjects (78 females and 58 males). Sensations reported after contact between the palmar hand surface and the thermal grill in grill mode, i.e. with alternating 20 and 40 °C tempered bars, exhibited a characteristic thermal profile. All 136 subjects reported sensations of “warm” or “hot” in the centre of the area in contact with the skin and 41 subjects (30%) described this sensation as painful (Table 2). All subjects (except three) reported “cold” mostly at the periphery of the contact surface (e.g. at the finger tips). The intensity of the cold sensation changed during grill stimulation in some subjects, and 10 subjects (7%) reported no “cold” at the end of the grill stimulation. Some 84 subjects (65%) described the entire sensation as “unpleasant” (Table 2). When being asked to report on additional qualities of the evoked perception, the descriptors burning, stinging and prickling were chosen by 57, 20 and 21 subjects (42, 15 and 15%), respectively (Table 2). Similar thermal grill data were obtained in the additional group of 40 subjects; the data are summarized in a table (see Additional file 1: Table S1).

**Table 2:** Sex differences in the sensations evoked by thermal grill stimulation (20/40 °C)

	♀ (*N* = 78)		♂ (*N* = 58)	
Sensation	*N*	%	*N*	%
Warm/heat	78	100.0	58	100.0
Cold	72	92.3	54	93.1
Unpleasantness	54	69.2	30	51.7*
Pain	30	38.5	11	19.0*
Burning	39	50.0	18	32.8*
Stinging	10	12.8	10	17.2
Prickling	9	11.5	12	20.7

Significant differences between sexes are marked by asterisks (Mann Whitney *U* test)

**Fig. 1:**
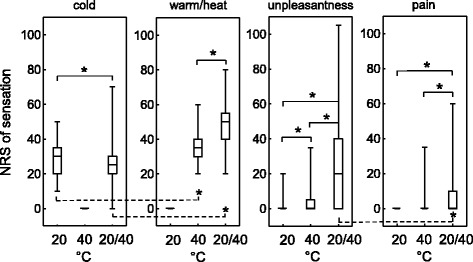
Numeric scale ratings (NRS) of thermal sensations (cold, warm/ heat), unpleasantness and pain evoked by placement of the right hand on the thermal grill for 20 s. For insets “cold” and “warm/ heat” hold “0 = neutral” and “100 = worst cold or worst warm/hot”, respectively. For insets “pain” and “unpleasantness” hold “0 = no pain or no unpleasantness” and “100 = worst pain or as unpleasant as can be imagined”, respectively. Three different thermal stimuli were tested: uniform 20 °C, uniform 40 °C and grill (bars tempered alternately at 20 and 40 °C) and each stimulus induced up to four different sensations. Ratings of all subjects (*N* = 136) are shown as medians with first and third quartiles (box) and range (whiskers, i.e. capped bars). Friedman’s ANOVA with post hoc testing using Wilcoxon’s signed-rank test was performed and significant differences are marked by asterisks

Pooled data of intensities of sensations arising from uniform thermal and grill stimuli are shown in Fig. 1 for all 136 subjects. In response to the uniform 40 °C stimulus all subjects reported the sensation of warm with a median intensity of 35 on the NRS. The uniform 20 °C stimulus evoked the sensation of cold in all subjects with a median intensity of 30 on the NRS. The thermal sensations of warm and cold evoked by the grill stimulus were rated with median intensities of 50 for warm (heat) and 30 for cold on the NRS. With respect to the intensity ratings, all four indicators “cold-induced cold sensation”, “warm-induced warm sensation”, “grill-evoked cold sensation” and “grill-evoked warm/heat sensation” differed significantly (*χ* ^2^ = 190.0, *p* < 0.001, Friedman’s ANOVA, *p* < 0.02, post hoc tests using Wilcoxon’s signed-rank test). Similarly, regarding the sensation of unpleasantness, the three stimulation conditions cold, warm and grill differed significantly (*χ* ^2^ = 85.5, *p* < 0.001, Friedman’s ANOVA, post hoc tests using Wilcoxon’s signed-rank test). Pain was felt by only one subject on exposure to the uniform warm stimulus (35 on the NRS). Under grill stimulation, pain was rated as less intense than unpleasantness (median value of 0 versus 20, *p* < 0.001, Wilcoxon’s signed-rank test).

Figure 1 shows numeric scale ratings of thermal sensations evoked by stimulation with the thermal grill.

### Sex differences in the TGI

Subjects were asked to report on the quality of the perception evoked by the thermal grill stimulation (20/40 °C interleaved). Table 2 lists the descriptors that the subjects could choose combined with the selection frequency for female and male subjects separately. Significant sex differences were observed regarding the sensations of “unpleasantness”, “pain” and “burning”. These sensations were described more often by females than males (*p* < 0.05, Mann-Whitney *U* test).

**Fig. 2:**
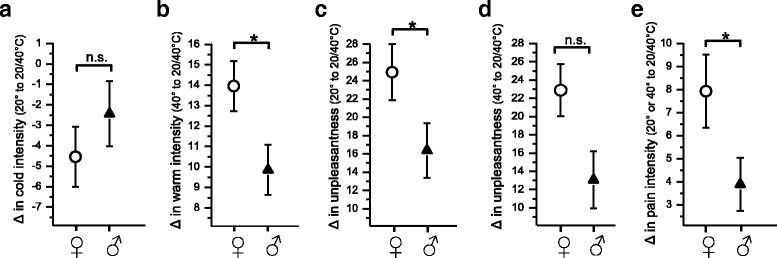
The change in intensity of different sensations (∆ values, means ± SEM﻿, panels **a**−**e**﻿) occurring by moving from the uniform temperature condition (20 or 40 °C) to the thermal grill condition (20 and 40 °C interleaved). To assess sex differences, Student’s *t* test for unpaired samples was performed and significant differences are marked by asterisks. In panel **e**, sensations evoked by the 20 or 40 °C stimulus were pooled because stimulation with uniform temperatures did not induce any pain sensation

Females and males did not differ with respect to the rated intensities of warm or cold sensation evoked by the uniform warm or cold stimuli (see Additional file 2: Figure S1). Additionally, both sexes rated the grill stimulus as warmer and more unpleasant than the uniform warm stimulus (*p* < 0.001, Wilcoxon’s matched pairs test, Bonferroni’s correction, Fig. 2). In order to analyse sex differences in thermal grill-evoked sensations, the changes of sensation intensities on moving from the uniform 20 or 40 °C to the grill mode (20/40 °C interleaved) were calculated. With respect to the change in warm intensity on moving from the uniform 40 °C to the grill mode, females showed greater changes than males. The difference in warm intensity between the two stimuli 40 °C and grill (*∆* values) was 14.0 ± 1.2 versus 9.7 ± 1.3 for female and male subjects, respectively, *p* < 0.05, Student’s *t* test, Fig. 2b). The difference in cold intensity was −4.5 ± 1.5 versus −2.5 ± 1.6 for female and male subjects (n.s., Student’s *t* test, Fig. 2a). The difference in the sensation of unpleasantness was 24.9 ± 3.1 (females) versus 16.3 ± 3.0 (males) for the two stimulation conditions 20 °C and grill (*p* < 0.05, Student’s *t* test, Fig. 2c) and 22.9 ± 2.9 (females) versus 13.0 ± 3.1 (males) for the two stimuli 40 °C and grill (*p* = 0.05, Student’s *t* test, Fig 2d). Sex differences in the sensation of unpleasantness, with females rating higher intensities than males, were also significant when including only the subjects who felt unpleasantness by thermal grill stimulation (data not shown). The difference in pain intensity between the two stimuli “uniform 20 or 40 °C” and grill was 7.9 ± 1.6 and 3.8 ± 1.2 for female and male subjects, respectively (*p* < 0.05, Student’s *t* test, Fig. 2e). However, when including the responders of pain only, the rated intensities did not differ between the two sexes (22.9 ± 2.6, *n* = 30 and 20.0 ± 2.8, *n* = 11 for females and males respectively, data not shown).

In summary, female subjects more often felt a burning sensation, unpleasantness and pain with a grill stimulus set at a 20/40 °C pattern than did males. In addition, females felt the grill stimulus, in comparison to the uniform cold or warm stimulus, as significantly warmer, less cold and more unpleasant than males.

Figure 2 shows sex differences in thermal grill-induced sensations.

### Sex differences in thermal thresholds

**Fig. 3:**
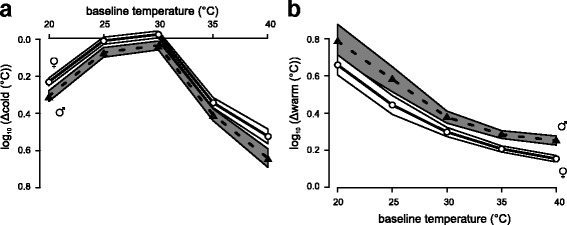
Cold (**a**) and warm (**b**) detection thresholds at different baseline (adaptation) temperatures for female (♀) and male (♂) participants. Means (log_10_(°C from baseline)) ± 95% confidence band of the means of the detection thresholds are shown. Mean threshold values of females are presented as open circles (confidence band: white area) and means of males are presented as filled triangles (confidence band: grey area)

To investigate sex differences in thermal detection and thermal pain threshold, a total of 9940 records from 1252 students (776 females and 476 males) were analysed. Thermal detection thresholds (CDT, WDT) were measured on the ventral surface of the forearm. Mean threshold values (°C from baseline) are shown in Fig. 3 and means ± SEM are summarized in Table 3. Both CDT and WDT were dependent on the baseline (adaptation) temperature, i.e. the starting temperature of the thermal ramp stimulus. The curve of WDT plotted against the baseline temperature (20–40 °C) showed a systematic decrease of WDT, with females showing significantly lower WDT values (smaller *∆* warm values in Fig. 3b) than males over the whole range of baseline temperatures tested (Tukey’s HSD, *p* < 0.05, Fig. 3b, Table 3). Within each sex group, WDT was significantly different in pairwise comparisons of neighbouring baseline temperatures, except for the pair 35 vs. 40 °C (Tukey’s HSD, *p* < 0.001). In contrast, the function of CDT vs. baseline temperature showed a completely different progression, namely that of an optimum curve with the highest CDT (smallest *∆* cold value in Fig. 3a) at 30 °C baseline temperature. CDT values (∆ values) were significantly higher at all other baseline temperatures tested (Tukey’s HSD, *p* < 0.001, Fig. 3a, Table 3). This effect was independent of sex. Females had significantly lower CDT values (smaller ∆ cold values) than males at all baseline temperatures tested (Tukey’s HSD, *p* < 0.05, Fig. 3a).

**Table 3:** Thermal detection thresholds at different baseline temperatures

	Sex	Baseline temperature (°C)				
		20	25	30	35	40
Warm threshold (°C)	♀	4.6 ± 1.07 *N* = 179	2.8 ± 1.06 *N* = 225	2.0 ± 1.03 *N* = 356	1.6 ± 1.02, *N* = 364	1.4 ± 1.02 *N* = 352
	♂	6.1 ± 1.11 *N* = 84	3.8 ± 1.08 *N* = 134	2.4 ± 1.04 *N* = 212	1.9 ± 1.02 *N* = 228	1.8 ± 1.03 *N* = 217
Cold threshold (°C)	♀	−1.7 ± 1.02 *N* = 338	−1.0 ± 1.02 *N* = 356	−0.9 ± 1.02 *N* = 375	−2.2 ± 1.03 *N* = 321	−3.3 ± 1.05 *N* = 253
	♂	−2.0 ± 1.03 *N* = 191	−1.2 ± 1.03 *N* = 227	−1.1 ± 1.03 *N* = 238	−2.6 ± 1.04 *N* = 202	−4.4 ± 1.06 *N* = 135

Thermal detection thresholds are listed as °C from baseline (mean ± SEM) for *N* subjects at different baseline temperatures

Figure 3 shows sex differences in cold and warm detection thresholds at different baseline temperatures.

CPT and HPT were measured over the thenar eminence starting at a baseline temperature of 32 °C. CPT for females was significantly higher (less cold) and HPT lower than for males (Tukey’s HSD, *p* < 0.01). The median CPT was 17.7 °C (*N* = 208) for females and 14.0 °C (*N* = 88) for males. The median HPT was 45.9 °C (*N* = 333) for females and 48.1 °C (*N* = 247) for males. In summary, females were more sensitive with respect to thermal detection and thermal pain thresholds than males.

**Fig. 4:**
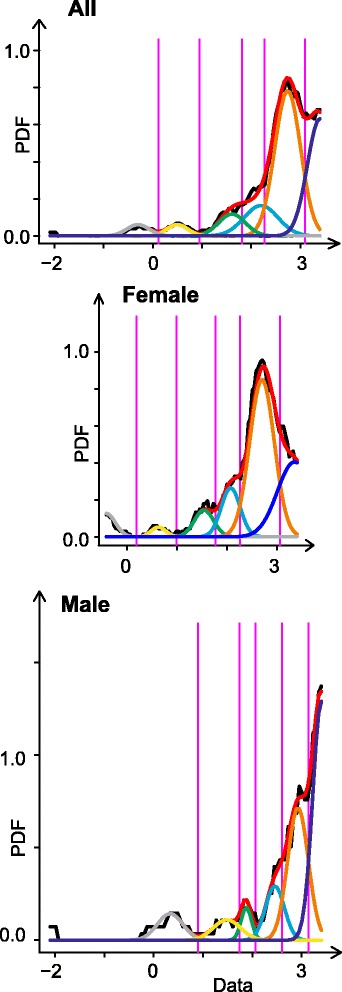
Gaussian mixture model of cold pain thresholds. The graphs display the data after rescaling for stimulus intensity and subsequent log transformation. The density distributions are presented as probability density function (PDF), estimated using a Gaussian mixture model (R-program AdaptGauss [30]). The optimum number of mixes was found to be M = 6. Black curve: PDF; red curve: Gaussian mixed model, Gaussians: grey = 1, yellow = 2, green = 3, cyan = 4, orange = 5, blue = 6; purple: Bayes boundaries of the Gaussians. ALL: *N* = 296 subjects, FEMALE: *N* = 208, MALE: *N* = 88

Analysis showed that the distribution of CPTs was clearly multi-modal. The distribution of the *N* = 296 log-transformed threshold data could be described with a Gaussian mixture model composed of M = 6 Gaussians (Fig. 4 and Additional file 3: Table S2). The first two Gaussians at 31.3 °C (grey) and 30.4 °C (yellow), after data was retransformed from the log domain to the threshold temperatures, represent the false responses of the subjects indicating CDT instead of CPT. The modes of the Gaussians #3, #4, #5 and #6 (Fig. 4: green, cyan, orange and blue curves) were obtained at 27.0, 23.0, 16.5 and 2.1 °C. The Gaussians #3, #4 and #5 showed significantly different modes between male and female subjects (Welch modified two-sample *t* test, *p* < 0.01; Additional file 3: Table S2).

Figure 4 shows a Gaussian mixture model of cold pain thresholds.

### Correlation of the TGI with thermal sensitivity

**Fig. 5:**
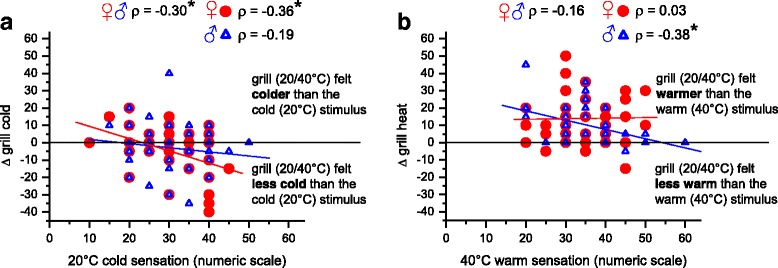
The correlation between ∆ grill cold, i.e. the change in cold intensity from the 20 °C to the thermal grill condition (20/40 °C) and the intensity of cold during the 20 °C condition (**a**). The correlation between the ∆ grill warm, i.e. the change in warm intensity from the 40 °C to the thermal grill condition (20/40 °C) and the intensity of warmth during the 40 °C condition (**b**). A positive number on the *y*-axis indicates an increase in cold or warm sensation during the thermal grill stimulation compared to the uniform cold (20 °C) or warm (40 °C) stimulus, respectively. A negative number indicates the opposite, i.e. a decrease. Data of male (*N* = 58) and female (*N* = 78) subjects are presented separately using different symbols and colours. As values of thermal intensity ratings were not normally distributed (Kolmogorov-Smirnov test), Spearman’s correlation coefficient *ρ* was calculated. *P* < 0.05 after Bonferroni’s correction was considered significant and this is indicated by asterisks

The sensation of warm evoked by the uniform warm stimulus correlated positively with the sensation of cold evoked by the uniform cold stimulus in both sexes (*ρ* = 0.26, *p* = 0.009 after Bonferroni’s correction, data not shown) demonstrating that the thermal sensitivities to warmth and coldness are associated. Figure 5 illustrates simple linear regression results using either warm stimulus-evoked warm sensation or cold stimulus-evoked cold sensation as the only independent variable: The increase in warm sensation evoked by grill stimulation (∆ grill heat, i.e. the difference in warm intensity between the two stimuli 40 °C and grill) correlated negatively with the warm stimulus-evoked warm sensation in males (*ρ* = −0.38, *p* = 0.006 after Bonferroni’s correction, Fig. 5b) whereas there was no significant correlation in females (*ρ* = 0.03, n.s.). Thus, in male subjects ∆ grill heat decreased with increasing sensitivity to warmth. ∆ grill cold, i.e. the difference in cold intensity between the two stimuli 20 °C and grill, decreased with an increasing cold stimulus-evoked sensation of cold (negative correlation with *ρ* = − 0.30, *p* = 0.001 after Bonferroni’s correction). This means that the higher the cold sensitivity of a subject the less cold was the sensation evoked by the grill stimulus. This effect was stronger in females than in males (*ρ* = − 0.36, *p* = 0.001 after Bonferroni’s correction) for females versus *ρ* = − 0.19, n.s. for males, Fig 5a).

Figure 5 shows correlations of the thermal grill-induced changes in thermal sensations with thermal sensitivity.

### Correlation of the TGI with thermal pain threshold

Consistent with the retrospective data analysis of QST measurements in 1.252 subjects, females in the group of 40 subjects showed significantly higher (less cold) CPT values than males (median CPT at the thenar eminence 10.4 for females versus 7.4 for males, *p* < 0.03, Mann Whitney *U* test, Additional file 1: Table S1). Regarding ∆ thermal pain threshold (HPT versus CPT), females showed significant lower values than males revealing a higher thermal pain sensitivity of females compared with males (*p* < 0.02, Student’s *t* test, Additional file 1: Table S1). The TGI obtained in the group of 40 subjects was similar to the TGI found in the group of 136 subjects, although sex differences were not significant in the small group (e.g. for ∆ grill cold *p* = *0.09*, Student’s *t* test, table in Additional file 1: Table S1).

**Fig. 6:**
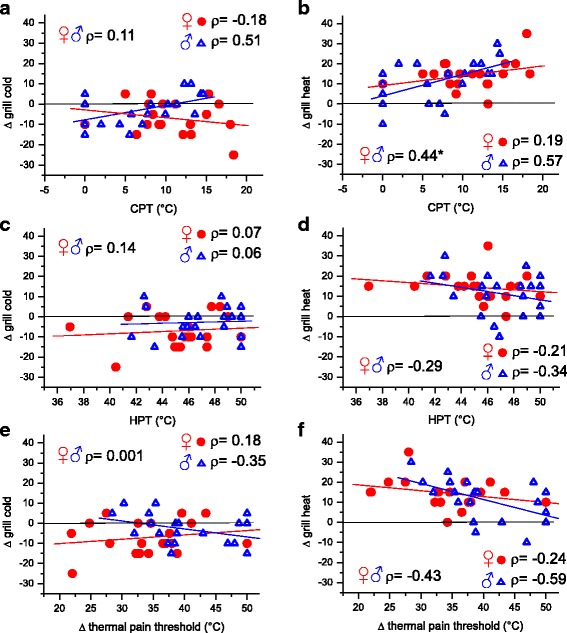
Linear regressions of ∆ grill heat (grill-induced increase in warm/heat sensation, panels **b**, **d** and **f**) and ∆ grill cold (grill-induced increase in cold sensation, panels **a**, **c** and **e**) with either CPT, HPT or ∆ thermal pain threshold (HPT versus CPT) as the only dependent variable. Data from male (*N* = 20) and female (*N* = 20) subjects are presented separately using different symbols and colours. As the thermal threshold values were not normally distributed (Kolmogorov-Smirnov test) Spearman’s correlation coefficient (*ρ*) was calculated to assess dependency between two variables. *P* < 0.05 after Bonferroni’s correction was considered significant and this is indicated by asterisks

Multiple regression analyses of ∆ grill heat were carried out using CPT, HPT and ∆ thermal pain threshold as the independent variables. Including all 40 subjects of both sexes showed that ∆ grill heat correlated significantly with CPT (*ρ* = 0.44, *p* = 0.03 after Bonferroni’s correction). Simply stated, within individual subjects, the higher, i.e. the less cold the CPT, the more intense the grill-induced increase of warm/heat sensation. Thus, females showing higher CPT than males showed a stronger grill heat sensation (Fig. 6b). Multiple regression analysis of ∆ grill cold with CPT, HPT and ∆ thermal pain threshold as the independent variables showed no significant dependency of ∆ grill cold with any of these variables. Sex differences in ∆ grill cold were found with respect to the slopes of the linear regressions. With both, increasing CPT and decreasing ∆ thermal pain threshold, ∆ grill cold decreased in females while it increased in males (Fig. 6a, e). This means that females described the grill stimulus as increasingly less cold while males described it as decreasingly less cold with higher cold pain sensitivity or higher thermal pain sensitivity, respectively.

Figure 6 shows correlations of the thermal grill-induced changes in thermal sensations with thermal pain thresholds.

## Discussion

The present study demonstrates that females show a stronger TGI than males, since females more often feel a burning sensation, unpleasantness and pain and describe the grill stimulus as significantly warmer and less cold than males. In addition, the study demonstrates that females show a higher thermal sensitivity and thermal pain sensitivity than males. The stronger TGI in females correlates with the higher sensitivity to cold and cold pain in females compared with males.

### Sex differences in the TGI and thermal sensitivity

In the present study thermal grill stimulation (20/40 °C interleaved) of the hand induced a unique perception including sensations of warmth, coldness, unpleasantness, burning, stinging, prickling and pain. Consistent with the literature, the present study shows the TGI to be very complex [10–23]. A novel finding in the present study was that the TGI is also sex-dependent. Regarding thermal sensitivity and thermal pain sensitivity, we also found sex differences in the present study. With respect to detection thresholds, our data are consistent with earlier studies [4, 7]. We found that the mean detection threshold of females and males differed by 0.2–0.4 °C, with females reporting higher (less cold) CDT values and lower (less warm) WDT values than males, indicating thermal sensitivity is higher in females than in males (see Table 3 and Fig. 3). The sex difference in WDT was more pronounced at low skin temperatures, e.g. 1.5 °C for WDT_20 °C_ and WDT_25 °C_ (see Fig. 3b), a new finding that reveals clear sex differences in warm detection threshold at slightly cool (25 °C) or cold (20 °C) skin temperatures thus implying sex differences in the complex processing of “cold” and “warm” inputs in thermal perception. For thresholds of cold pain and heat pain, females showed lower HPT and higher (less cold) CPT values in the present study which is consistent with the literature, albeit for CPT data published findings are somehow contradictory [2–4, 6, 8].

With respect to the distribution of CPT data, we found a sex-dependent multimodal distribution in the present study that is similar to the CPT data distribution published recently by Lötsch and colleagues [31]. Fitting a Gaussian mixture model to the log-transformed CPT data revealed three Gaussian distributions with modes at 23, 17 and 2 °C (see Fig. 4). According to Lötsch and colleagues [31], the localization of the first and second Gaussians may be interpreted as reflecting the contribution of the TRPM8 receptor that starts to respond at 24 °C [32] and the TRPA1 receptor that starts to sense cold at 17 °C [33]. Sex differences were found for these Gaussians in the present study indicating sex-dependent receptor characteristics at the skin area where the cold stimuli had been applied. For the Gaussian with mode at 2 °C, a sex-dependent difference of response probability was found (female 15%, male 32%, respectively (see Additional file 3: Table S2) indicating that other temperature-sensing receptors, e.g. TRPC5, KCNK2, ASIC2 or ASIC3, might show sex-dependent spatial densities in the skin as well. The mode at 27 °C may be seen as the earliest response due to an uncomfortable cold stimulus and the modes at 31.3 and 30.4 °C as false responses of untrained students who indicated the CDT instead of the CPT.

### Thermal sensitivity as a function of the baseline temperature

Our retrospective analysis of sex-dependent thermal threshold data provides the novel finding that warm and cold detection thresholds are seen to be affected by the baseline (adaptation) temperature. Studies to date have addressed thermal detection thresholds at baseline temperatures around the neutral/comfort zone, usually 32 °C, which is approximately the mean skin temperature at standard ambient temperature [24, 25]. Our data demonstrate that, independent of sex, the CDT as a function of the baseline temperature has the form of an optimum curve with the optimum in the range 25–30 °C. In humans, innocuous skin temperatures of cold are signalled by cold-sensitive Aδ fibres [13, 34]. Recently, a micro-neurography study in humans has shown that the response rate of an Aδ fibre to a staircase cold stimulation has the form of an optimum curve with the maximum response rate at 26 °C baseline temperature and lower response rates at lower or higher baseline temperatures (see Fig. 7 in [34]. Hence, our CDT data (see Fig. 3a) might be explained by Aδ fibre activation. In addition, the activity of C2 fibres, a population of C fibres responding to warming and innocuous cooling [34] is likely to play a role. C2 fibre activity is inhibited by Aδ fibre input; when the Aδ fibre input is blocked experimentally, innocuous cooling becomes painful [35, 36], probably by disinhibition of C2 fibre activity. Thus, the interplay between Aδ and C2 fibre activity during skin cooling is likely to depend on the baseline (adaptation) temperature of the skin. In contrast, the curves of WDT vs. baseline temperature show a hyperbolic form with the lowest WDT at 40 °C and the highest at 20 °C (see Fig. 3b) confirming data of early studies [37, 38]. According to the literature, warm fibres in primates respond to warm stimuli above 30 °C only [38–42], conversely there is no evidence for warm receptor activation by warm stimuli at baseline temperatures below 30 °C. Thus, the mechanism of warm detection at cool skin temperatures remains unclear. At cool skin temperatures, Aδ and C2 fibres are activated. During subsequent warming of the skin, activation decreases for Aδ fibres and increases for C2 fibres. This may lead to a sensation of declining cold and thus to the subjects’ reaction to denote their WDT.

### Correlation of the TGI with cold sensitivity and cold pain sensitivity

A model proposed to explain the TGI, albeit excluding the non-painful sensations, indicates that thermal grill stimulation with interleaved warm and cold stimuli reduces the cold signal that is responsible for inhibiting the activity in central multi-modal neurons responsive to heating, pinch and cold (HPC), hence leading to disinhibition and consequently by increasing the magnitude of the HPC signal to pain [10]. The assumption that a strong cold signal is jointly responsible for the TGI is supported by the recent finding that topical application of menthol, an activator of the cold signal, leads to an enhanced grill-evoked heat sensation [19], probably by enhancing the disinhibition of the HPC signal. Consistent with this is the finding of the present study that both cold and cold pain sensitivity are related to the intensity of the TGI (see Figs. 5 and 6), e.g. the stronger the individual sensation of cold under the uniform cold stimulus, the larger was the decrease of cold sensation under grill conditions in the present study (see Fig. 6a). In contrast, warm and heat pain sensitivity were not related to the thermal grill-evoked sensations in the present study showing that the “warm” input is less important for the TGI (see Fig. 6b).

Recent publications have reported that higher sensitivity to cold and heat pain is associated with a stronger TGI, e.g. stronger sensations of unpleasantness and pain [18, 43]. The reverse effect has been found in studies with patients with psychiatric disorders showing that a lower sensitivity to cold and heat pain is accompanied by a less intense TGI compared with controls [16, 44]. Thus, sex differences in the thermal grill sensations are assumed to be related to sex differences in heat and cold pain. Our correlation analysis (see Figs. 5 and 6) shows that the sex-dependent sensitivity to cold and cold pain is responsible for the sex-dependent TGI. It is primarily the decrease of cold sensation under grill conditions that differs between the two sexes. In the present study, the reduction of cold sensation under grill stimulation increased with higher CPT in females but decreased in males (see Fig. 6a). Thus, the females’ higher sensitivity to cold pain is related to the grill-evoked sensation of less cold in females compared with males. In addition, the present study shows that the higher the non-painful cold sensitivity of a subject the less cold was the grill-evoked sensation and this effect was stronger in females than in males (see Fig. 5a and Discussion above). The females’ grill-evoked sensation—warmer and more unpleasant (and if so burning and painful) than the males’ grill-evoked sensation—is supported by the females’ sensation of less cold, which is dependent on the sensitivity to cold and cold pain.

### Limitations

It has to be taken into account that the retrospective data analysis was performed on QST data collected by self-performed measurements by students who were only informed but not trained in the measurement procedure. This fact may reduce the validity of the test. When comparing thermal pain threshold of the retrospectively analysed data with thermal pain threshold obtained in additional QST measurements that were carried out by an investigator in a group of students who were trained in QST measurements, we found similar values for HPT and colder values for CPT in trained versus untrained subjects (see “Sex differences in thermal thresholds” and Additional file 1: Table S1). The less cold CPT in untrained than in trained subjects may be due to the untrained subjects indicating the CDT instead of the CPT in the experiment (see Fig. 4 and Additional file 3: Table S2). Another possibility is that trained subjects may wait a little longer before “confirming” their sensation of cold pain than untrained subjects. Regarding sex differences in CPT, females and males differed independent of training. A limitation of the present study is the fact that a female investigator tested the TGI and the thermal pain threshold in the small cohort of 40 subjects. This may bias psychophysical outcomes in relation to sex. For painful stimuli, male subjects reportedly show weaker responses when the investigator is female rather than male [45, 46]. Another limitation of the present study is the fact that different stimulation sites were used to determine thermal detection and thermal pain threshold in the retrospectively analysed QST measurements (ventral surface of the forearm near the wrist and thenar eminence, see 2.2). However, the stimulation sites including the hand stimulated in the thermal grill experiments are unlikely to differ markedly; in an earlier study, thermal detection and pain threshold and the TGI were found to be similar for the volar forearm and the palm of the hand [19].

## Conclusions

Our study provides further evidence for a strong interaction between the thermoreceptive and nociceptive systems. The new aspect of the retrospective data analysis of our study is the finding that thermal detection threshold not only differs between the two sexes but is also dependent on the baseline temperature. A specific progression of an optimum curve was found for the function relating CDT to baseline temperature with the highest sensitivity of cold detection around the basal skin temperature implying a complex processing of “cold” and “warm” inputs in thermal perception. The multimodal distribution of the cold pain thresholds indicates sex-dependent differences in response characteristics and spatial densities of the cold receptors TRPA1 and TRPM8 in the skin. Our findings regarding the TGI, first, that the TGI was more pronounced in females and, second, that the intensity of the illusion correlated with the subjects’ sensitivity to cold and cold pain leads to the assumption that sex differences in the cold-related thermoreception are responsible for sex differences in the TGI. For further investigation, it would be of interest to test the sex dependency of the TGI under a strong activation of the cold signal, e.g. by the application of cold receptor agonists corresponding to TRPA1 and TRPM8.

## Additional files

Additional file 1: Table S1. Thermal pain threshold and thermal grill data obtained in 40 subjects. These data were used for correlation analysis of thermal pain threshold and the TGI. (DOCX 38 kb)

Additional file 2: Figure S1. Numeric scale ratings (NRS) of sensations (cold, warm/heat, unpleasantness, pain) evoked by stimulation with three different thermal stimuli: uniform 20 °C or 40 °C or grill mode (bars tempered alternately at 20 °C and 40 °C). Ratings (medians with first and third quartiles (box) and range (whiskers)) are presented sex-dependently, in red for female (*N* = 78) and in blue for male (*N* = 58) subjects. Significant differences between sexes are marked by asterisks (Mann Whitney *U*-Test). (PDF 100 kb)

Additional file 3: Table S2. Characteristic values of the Gaussian mixture model for the cold pain thresholds. After rescaling the data for stimulus intensity and subsequent log transformation the probability density function (PDF) was estimated using a Gaussian mixture model with six modes (R-program AdaptGauss [30]). (DOCX 13 kb)

## Abbreviations

CDT: Cold detection threshold
CPT: Cold pain threshold
HPC: Heating, pinch, cold
HPT: Heat pain threshold
IQR: Interquartile range
NRS: Numeric rating scale
QST: Quantitative sensory testing
SEM: Standard error of the mean
TGI: Thermal grill illusion
WDT: Warm detection threshold
