# Supplementary material for: Sex differences in thermal detection and thermal pain threshold and the thermal grill illusion: a psychophysical study in young volunteers
Source: Biol Sex Differ. 2017 Sep 1;8:29. doi: 10.1186/s13293-017-0147-5 (PMC5579939; doi:10.1186/s13293-017-0147-5)
Supplement: Supplementary file 1 — Thermal pain threshold and thermal grill data obtained in 40 subjects. These data were used for correlation analysis of thermal pain threshold and the TGI. (DOCX 38 kb) [file 13293_2017_147_MOESM1_ESM.docx]

**Additional file 1 Table S1**

**Thermal pain threshold and thermal grill data used for correlation analysis.**

♀ (*N*=20) ♂ (*N*=20)

**Thermal pain threshold (median)**

Heat pain threshold (HPT) 45.5 °C 46.8 °C p=0.07

Cold pain threshold (CPT) 10.4 °C 7.4 °C p=0.03

mean of HPT versus CPT 34.3 °C 39.9°C p=0.03

**Sensations evoked by thermal grill stimulation (20/40°C) in *N* (% of) subjects**

Warm/heat 20 (100) 20 (100)

Cold 17 (85) 19 (95)

Unpleasantness 15 (75) 10 (50)

Pain 5 (25) 3 (15)

Burning 11 (55) 8 (40)

Stinging 2 (10) 2 (10)

Prickling 3 (15) 3 (15)

**Change in intensity of different sensations occurring by moving from the uniform temperature condition to the thermal grill condition (**Δ **values,** **means ± SEM)**

Δ in heat (40° to 20/40°C) 14.5 ± 1.6 11.5 ± 2.3

Δ in cold (20° to 20/40°C) -7.0 ± 1.8 -2.8 ± 1.7

Δ in unpleasantness (20° to 20/40°C) 27.5 ± 5.6 22.8 ± 6.3

Δ in unpleasantness (40° to 20/40°C) 27.3 ± 5.3 22.0 ± 6.5

Δ in pain (20 or 40° to 20/40°C) 10.3 ± 4.5 7.8 ± 4.2

Sex differences in thermal pain threshold are calculated using the Mann Whitney *U*-Test.
