# Supplementary figures and images for: Sex differences in thermal detection and thermal pain threshold and the thermal grill illusion: a psychophysical study in young volunteers
Source: Biol Sex Differ. 2017 Sep 1;8:29. doi: 10.1186/s13293-017-0147-5 (PMC5579939; doi:10.1186/s13293-017-0147-5)

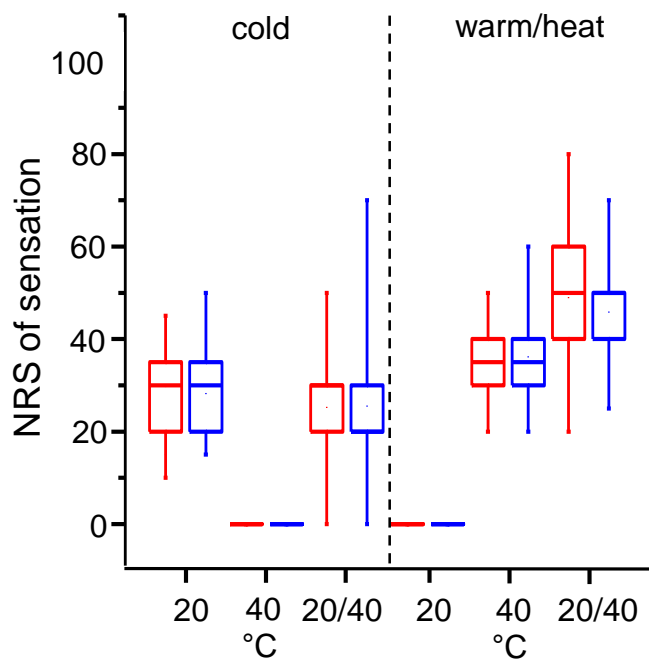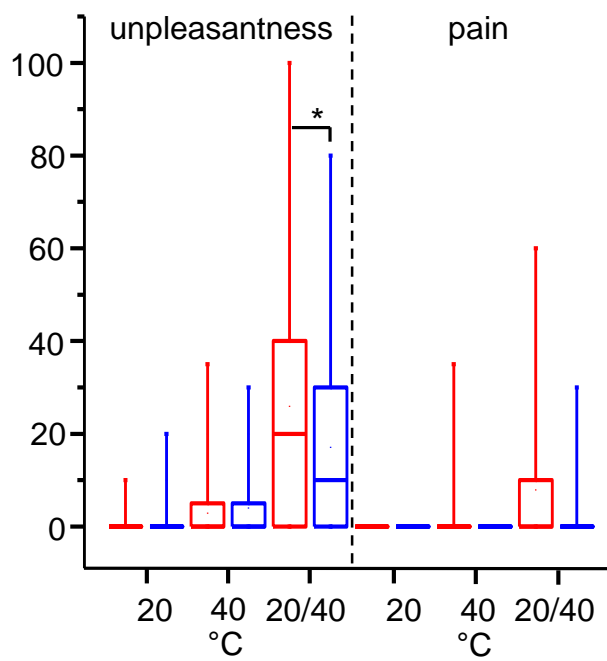

Supplement: Supplementary file 2 — Numeric scale ratings (NRS) of sensations (cold, warm/heat, unpleasantness, pain) evoked by stimulation with three different thermal stimuli: uniform 20 °C or 40 °C or grill mode (bars tempered alternately at 20 °C and 40 °C). Ratings (medians with first and third quartiles (box) and range (whiskers)) are presented sex-dependently, in red for female (N = 78) and in blue for male (N = 58) subjects. Significant differences between sexes are marked by asterisks (Mann Whitney U-Test). (PDF 100 kb) [file 13293_2017_147_MOESM2_ESM.pdf]
