# Supplementary material for: Sex differences in thermal detection and thermal pain threshold and the thermal grill illusion: a psychophysical study in young volunteers
Source: Biol Sex Differ. 2017 Sep 1;8:29. doi: 10.1186/s13293-017-0147-5 (PMC5579939; doi:10.1186/s13293-017-0147-5)
Supplement: Supplementary file 3 — Characteristic values of the Gaussian mixture model for the cold pain thresholds. After rescaling the data for stimulus intensity and subsequent log transformation the probability density function (PDF) was estimated using a Gaussian mixture model with six modes (R-program AdaptGauss [30]). (DOCX 13 kb) [file 13293_2017_147_MOESM3_ESM.docx]

**Additional file 3 Table S2**

**Characteristic values of the Gaussian Mixture Model for the cold pain thresholds.**

|  | Gaussian mode | | | | | |
| --- | --- | --- | --- | --- | --- | --- |
| **All** (N = 296) | 1 | 2 | 3 | 4 | 5 | 6 |
| Mean | -0.3016 | 0.4980 | 1.6105 | 2.2008 | 2.7439 | 3.399 |
| SD | 0.2307 | 0.2123 | 0.2709 | 0.3444 | 0.2625 | 0.2541 |
| Relative Weight | 0.0352 | 0.0327 | 0.0805 | 0.1421 | 0.5157 | 0.4025 |
| Upper Bayes boundary | 0.1142 | 0.9526 | 1.8173 | 2.2765 | 3.0987 |  |
| Percentage data below upper Bayes boundary | 3.0 | 6.8 | 14.6 | 25.4 | 79.8 |  |
| Mean in °C | 31.3 | 30.4 | 27.0 | 23.0 | 16.5 | 2.1 |
|  |  |  |  |  |  |  |
| **Female** (N = 208) |  |  |  |  |  |  |
| Mean | -0.4113 | 0.6311 | 1.5437 | 2.0782 | 2.7044 | 3.3535 |
| SD | 0.1819 | 0.1446 | 0.1938 | 0.1894 | 0.2460 | 0.3414 |
| Relative Weight | 0.0579 | 0.0194 | 0.0704 | 0.1258 | 0.5245 | 0.3472 |
| Upper Bayes boundary | 0.1913 | 0.9901 | 1.7727 | 2.2604 | 3.0678 |  |
| Percentage data below upper Bayes boundary | 2.9 | 5.3 | 13.5 | 26.0 | 85.1 |  |
| Mean in °C | 31.3 | 30.1 | 27.3 | 24.0 | 17.1 | 3.4 |
|  |  |  |  |  |  |  |
| **Male** (N = 88**)** |  |  |  |  |  |  |
| Mean | 0.3339 | 1.4824 | 1.8769 | 2.4564 | 2.9242 | 3.399 |
| SD | 0.2555 | 0.2759 | 0.1042 | 0.1901 | 0.2085 | 0.1759 |
| Relative Weight | 0.0919 | 0.0761 | 0.0465 | 0.1396 | 0.3748 | 0.5686 |
| Upper Bayes boundary | 0.9024 | 1.7366 | 2.0648 | 2.6025 | 3.1372 |  |
| Percentage data below upper Bayes boundary | 10.2 | 14.8 | 21.6 | 36.4 | 68.2 |  |
| Mean in °C | 30.6 | 27.6 | 25.5 | 20.3 | 13.4 | 2.1 |

After rescaling the data for stimulus intensity and subsequent log transformation the probability density function (PDF) was estimated using a Gaussian mixture model with six modes (R-program AdaptGauss [30]).
